# 

**Published:** 1890-01

**Authors:** 


### WEST TEXAS DISTRICT MEDICAL ASSOCIATION.

### Third Quarterly Meeting.

SAN ANTONIO, January 15, 1890.

DEAR DOCTOR:—You are cordially invited to attend the third quarterly session of the West Texas Medical Association, to be held in San Antonio, Wednesday, January 29th. Three meetings will be held; at 10 a. m., 3 p. m., and 7:30 p. m. Papers will be presented by the following gentlemen:

Dr. F. Herff: Cerebral localization in surgery.

Dr. E. Cross: Subinvolution.

Dr. W. P. Flemming: Cancrum Oris.

And a paper by W. H. Way, of Austin.

Following the night meeting, an oyster supper will be given at the Elite Restaurant. Visiting gentlemen will be guests of the Association. Very respectfully,

D. BERRY, M. D.

WHO WAS YOUR GREAT GRANDFATHER ?—The Detroit Journal desires to receive, by postal card, the address of all living male and female descendants of Revolutionary officers and soldiers of 1776, and, when possible, the name and state of the an cestor. Wonder if W. H. Breasley, proprietor of the Detroit Journal, is contemplating a raid upon the national treasury?

